# The Phosphorus and the Vascular Calcification in ESRD between Old Adventures and New Horizons

**DOI:** 10.4061/2011/716526

**Published:** 2012-02-27

**Authors:** Biagio Raffaele Di Iorio, Markus Ketteler, Domenico Russo, Angela Wang

**Affiliations:** ^1^Division of Nephrology, “A. Landolfi” Hospital, I-83029 Solofra (AV), Italy; ^2^Division of Nephrology, Klinikum Coburg, 96450 Coburg, Germany; ^3^Department of Nephrology, School of Medicine, University Federico II, Via Pansini 5, I-80131 Napoli, Italy; ^4^University Department of Medicine, Queen Mary Hospital, University of Hong Kong, 102 Pok Fu Lam Road, Hong Kong

Phosphate plays a central role in the pathophysiology of CKD-MBD and the progression of chronic kidney disease (CKD) and contributes to the disproportionate cardiovascular risk faced by patients with CKD. Adaptation of nephrons attempting to preserve phosphate homeostasis requires endocrine tradeoff that fuel adverse events of hyperphosphatemia in CKD—secondary hyperparathyroidism, calcium and vitamin D derangements, vascular calcifi cation, and metabolic bone disorder.

In fact, in advancing CKD there is a multitude of biochemical, physiological, and clinical alterations, and mechanistic understanding of secondary hyperparathyroidism, vascular calcification, and regulation of phosphate metabolism in CKD has advanced significantly in the past five decades. The principal hormones that regulate renal phosphate handling are parathyroid hormone (PTH), which is produced by the parathyroid gland, and fibroblast growth factor (FGF)-23, which is produced by osteocytes and osteoblasts in bone. In healthy individuals, increasing serum phosphate concentration induces secretion of PTH and FGF-23, and Kloto decline.

It has been established (and now accepted by nephrologists) that observational studies strongly suggest that phosphorus is associated with cardiovascular risk, and definitive prospective animal studies are supportive. However, prospective studies demonstrating that modulation of the putative risk factor affects clinical outcomes are lacking, and phosphorus, as yet, does not qualify as a cardiovascular risk factor.

This special issue provides an identification of the complex mechanisms that determine the phosphorus and calcium metabolism in CKD-3 and CKD-4; a verify the association of serum phosphate and related factors in ESRD-related vascular calcification. 

It has also addressed mineral metabolism in patients with CKG in the Era of KDIGO Guidelines, the effect of vitamin D on clinical outcomes in chronic kidney disease, and the effect of paricalcitol on vascular calcification and cardiovascular disease in uremia. 

Finally, were also discussed the effect of the use of non-calcium-phosphate binders in the control of vascular calcifications; the clinical significance of FGF-23 in patients with CKD, the the relationship between arterial stiffness and vascular damage; the treatment of severe metastatic calcification and calciphylaxis in dialysis patients; the clinical impact of hypercalcemia in kidney transplant patients.

*Biagio Raffaele Di Iorio* *Biagio Raffaele Di Iorio* *Markus Ketteler* *Markus Ketteler* *Domenico Russo* *Domenico Russo* *Angela Wang* *Angela Wang*

